# Antibiotic therapy alone does not have a high success rate in cases of unexpected positive cultures in intraoperative samples from hip and knee prosthesis revision

**DOI:** 10.1186/s12891-020-03799-w

**Published:** 2020-11-28

**Authors:** Bernd Fink, Michael Schlumberger

**Affiliations:** 1 Department for Joint Replacement, Rheumatoid and General Orthopaedics, Orthopaedic Clinic Markgröningen, Kurt-Lindemann-Weg 10, 71706 Markgröningen, Germany; 2 Orthopaedic Department, University Hospital Hamburg-Eppendorf, Martinistrasse 52, Hamburg, 20251 Germany

**Keywords:** Periprosthetic joint infection, Positive culture intraoperative, Antibiotic treatment

## Abstract

**Background:**

Unexpectedly positive bacterial cultures during prosthesis revision surgery still occur on occasion despite good preoperative diagnostics. In such cases a six-week antibiotic therapy without further surgical intervention is recommended. The aim of this study was to find out how successful this procedure is.

**Methods:**

In a study of 508 patients, who required revision surgery of total hip (THA, *n* = 231) or knee arthroplasties (TKA, *n* = 277) because of component loosening, biopsy was carried out before their surgery. The collected tissue samples (5) from the biopsy and the revision surgery procedure itself were analyzed according the criteria of the International Consensus Meeting (ICM). Tests revealed 11 patients (7 THA, 4 TKA) with unexpectedly positive bacterial cultures from tissue samples obtained during the revision surgery due to false negative preoperative diagnostic results. These 11 patients were treated with 6 weeks antibiotic therapy and examined with a follow-up of at least 2 years (42.2 ± 16.5 months).

**Results:**

Five patients (2 TKA, 3 THA) became reinfected, resulting in a success rate of 54.5%.

**Conclusion:**

Antibiotic therapy alone of an unexpected positive intraoperative bacterial culture in prosthesis revision surgery seems to be less successful than previously assumed.

## Background

Periprosthetic joint infection (PJI) is a severe complication of joint replacement surgery, with an incidence ranging between 1 and 2% after primary and between 2 and 6% after revision arthroplasty [1–3]. However, some studies report PJI to be the most common cause for revision in the first 5 years following primary arthroplasty [1, 4–6]. The accuracy of the preoperative diagnosis of possible infection becomes especially important in cases of loosened and painful joint endoprostheses because the presence of a PJI would result in significant changes to the subsequent therapeutic procedures [7, 8].

However, despite all efforts of preoperative diagnostic procedures prior to prosthesis revision surgery, negative preoperative results can occasionally be followed by positive bacterial cultures of several tissue samples obtained during the revision surgery. Thus, the preoperative results can then be described as false negatives and, by definition, a periprosthetic infection can be declared. This situation is called “positive intraoperative culture” or “type 1 infection” in the most commonly used classification by Tsukayama et al. [9]. In the few, mostly older studies, the incidence of “type 1 infections” in prosthesis revisions is reported to be between 1.6 and 29.2% depending on the quality of the preoperative infection diagnostic procedures [9–13]. The recommendation is to treat this “type 1 infection” with a six-week administration of antibiotic without any further surgical intervention [9–13]. The chances of success of this procedure are stated to be between 81 and 100% in the few published reports in the literature [10–12, 14]. However, all the published reports are older and at that time preoperative diagnostic procedures were not the norm. The diagnostic procedures for detecting PJI were started intraoperatively at that time by taking culture samples during revision surgery [10–12, 14]. In addition, the current criteria for defining a periprosthetic infection were not available for application at that time [10–12, 14] and sometimes an insufficient number of intraoperative tissue samples (less than 5) were obtained [12, 15]. Therefore, it cannot be ruled out that some of the patients in those early studies were wrongly classified as PJI due to false positive intraoperative diagnostics and therefore a falsely high rate of success was determined for the sole antibiotic therapy of “type 1 infections”.

Moreover, in cases of true PJI, when diagnostic procedures are initiated during revision surgery, the evidence of a bacterial infection in the intraoperative samples only appears after several days of cultivation (up to 14 days depending on the microorganism) [16, 17]. Therefore, any bacteria present in situ during this period would have had time to form a biofilm on the revision implant. Even though formation of a mature biofilm takes around 4 weeks, susceptibility of the bacteria to antibiotics is reduced within the first 2 weeks of infection [18, 19]. Therefore, a high success-rate of antibiotic treatment alone in these cases seems to be questionable. In addition, in a similar situation of two-stage septic revisions, where positive bacterial cultures arise from tissue samples taken at the second stage of reimplantation, antibiotic therapy alone has been reported to be associated with a significantly higher reinfection rate of 45.5 and 45.8% [20, 21].

It therefore remains unclear whether, taking into account preoperative diagnostic procedures and modern PJI criteria, a high probability of success can still be expected with 6 weeks of antibiotic therapy alone in the event of unexpected intraoperative positive cultures in several samples (type 1 infection).

Therefore, the aim of this study was to assess the ability of antibiotic therapy to treat unexpected positive cultures that arose during aseptic revision arthroplasty. The results were used to test our hypothesis that 6 weeks of antibiotic therapy alone do not have a sufficient success rate in type 1 infection.

## Methods

A prospective study investigated a continuous series of 508 patients (253 female, 255 male) who required revision surgery of total hip (THA, *n* = 231) or knee arthroplasties (TKA, *n* = 277) because of component loosening [22]. Out of this 178 cases (113 THA, 65 TKA) had periprosthetic joint infection (PJI), as classified with the bacteriologic, histologic and serologic analyses at revision surgery using the ICM-2018 criteria [23].

Before revision surgery all 508 patients underwent a diagnostic intervention that involved blood C-reactive protein (CRP)-analysis and aspiration of the joint as well as biopsy of the periprosthetic tissue according to the methods already described in previous publications [22, 24, 25].

Briefly, both joint aspiration and biopsy techniques were carried out under sterile conditions in the operating theatre under general anaesthesia. At the hip the aspiration was performed using an antero-lateral approach under image intensifier control as described by Kilcoyne et al. [26]. At the knee the aspiration was performed at the suprapatellar recessus. For optimum results the harvested fluid was immediately injected into vials containing BD BACTEC-PEDS-PLUS/F-Medium (Becton Dickinson, Heidelberg, Germany) [27]. The biopsies were obtained using arthroscopic biopsy forceps introduced via a small antero-lateral approach at the hip under image intensifier control, and an antero-lateral approach at the knee (as used in arthroscopic surgery) and were taken from the periprosthetic tissue in five different areas, close to the prosthesis. Afterwards, five tissue samples were obtained for histological examinations. Prophylactic peri-operative antibiotics as a single dose of cephalosporin (2 g of Cefazoline) were administered once all samples had been obtained.

During the revision surgery itself samples were taken from 5 different areas of the periprosthetic membrane. In addition, five samples from the periprosthetic connective tissue membrane associated with the loosened prosthesis were obtained for histological assessment. In the aseptic expected revision surgeries irrigation with antiseptic solution (Lavasurge = Ringer solution with 0.04% Polihexanid, B. Braun, Melsungen, Germany) was used. In septic revision additionally a second antiseptic solution (Octenisept = 0.1% Octenidindihydrochlorid + 2% Phenoxyethanol, Schülke & Mayr GmbH, Norderstedt, Germany) was used for minmum 3 min and than washed out.

The biopsy samples and the tissue samples of the revision surgery were each placed in sterile tubes and transferred together with the aspirated fluid to the microbiological laboratory within an hour of sampling. Patient specimens were processed immediately after arrival at the laboratory. PEDS culture vials were treated with Fastidious Organism Supplement (FOS) (Becton Dickinson, Heidelberg, Germany), and incubated using the BD BACTEC 9050 automatic blood culture system (Becton Dickinson, Heidelberg, Germany). Cultures were discontinued and declared negative if no growth was reported after 14 days according to Schäfer et al. [17]. For cultivation tissue specimens were thoroughly minced under sterile conditions. Aerobic and anaerobic culture and gram staining was performed with all tissue suspensions. Media were checked daily for bacterial growth. Broths that remained clear were incubated for 14 days until the specimen was declared negative as described by Schäfer et al. [17]. Turbid broths were subcultured onto appropriate agar plates. Microorganisms were identified by standard microbiological procedures including biochemical characterization with the API system (BioMerieux, Nuertingen, Germany) in case of anaerobic strains or anaerobic bacterials. Antibiotic susceptibility testing was performed by disk diffusion or dilution methods according to the Clinical and Laboratory Standards Institute (CLSI) guidelines. In all other cases we used Vitek II (BioMerieux, Nuertingen, Germany) for identification and antibiotic susceptibility testing.

The results were analysed according to the ICM-2018-Definition [23] whereby a synovial membrane sample was regarded as positive when at least one of the following conditions had been fulfilled:

1. Demonstration of the same pathogen in at least two of the samples.
2. Demonstration of a pathogen in at least one sample and demonstration of at least five neutrophilic polymorph leukocytes in five high power field (× 400) in the associated histological preparation and an elevated CRP-value (> 10 mg/L) as described in the ICM-2018-Definition [23].

The presence of bacteria in only one sample without any histological confirmation was regarded as a result of contamination during the sampling procedure or during the incubation period, in accordance with Virolainen et al. [28].

The data of this group and the value for this diagnostic procedure (biopsies and its combination with aspiration and blood CRP-analysis) of these 508 patients was previously published and showed a sensitivity of 93.8%, a specificity of 97.3%, a positive predictive value (PPV) of 94.9%, a negative predictive value (NPV) of 96.7%, and an accuracy of 96.1% [22]. The mean age of the 508 patients was 68 ± 10 (30–87) years. Revision surgery was carried out 38.6 ± 38.1 months (3–210 months) after primary implantation.

Eleven of the 508 patients (2.2%) (7 THA and 4 TKA) had unexpected PJI because of positive intraoperative cultures at revision surgery in at least two tissue samples (out of 5 samples) and pre-operative false-negative diagnostics using C-reactive protein, aspiration and tissue biopsy for culture analysis and applying the ICM 2018-Criteria [23]. In 10 of these 11 patients the culture analysis of the biopsy was negative and once in 1 out of 5 samples the cultivation was positive, but was rated as contamination (case 6, Table 1). These were 5 females and 6 males with an age of 60.1 ± 13.3 years (29–80 years) (Table 1). Nine had loosening of both implant components, one patient with TKA required revision because of chronic joint stiffness, and one patient had a revision of only the hip stem because of loosening. All patients received 6 weeks of antibiotic treatment after the result of intraoperative sample cultures were found to be positive. The patient with just stem revision (case 10, Table 1) also received a cup revision to complete the exchange of all components, followed by the antibiotic treatment.

**Table 1:** Description of the eleven patients with unexpected positive intraoperative culture

Pat.	Gen-der	BMI	ASA	CCI	Joint	Histo-ry Joint	Number previous opera-tions at the joint including arthropl.	CRP Pre-biopsy (mg/L)	WBC count in synovia (cells/μL)	Micro-biology Biopsy	Histology Biopsy	ICM-Score pre-op	Micro-biology Revision	Histo-logy Re-vision	ICM-Score after revi-sion	Antibiotic Therapy	Re-infection after months
1	male	25.4	2	0	TKA	Loose-ning after 6 years	1	21	1360	neg.	neg.	2	Staph. hominis (3/5)	neg.	major + 2	Flucloxacillin + Rifampicin 2 weeks then Levofloxacin + Rifampicin 4 weeks	36 months with Staph. hominis
2	fem.	28.1	2	0	TKA	Revi-sion after 3 years because of arthro-fibrosis	3	< 5	800	neg.	neg.	0	Staph. epidermidis (3/5) Staph. hominis (1/5)	neg.	major	Vancomycin + Rifampicin 2 weeks then Levofloxacin + Rifampicin 4 weeks	no
3	male	26.3	2	0	TKA	Loose-ning after 10 years	1	< 5	630	neg.	neg.	0	Staph. hominis (2/5) Staph. epidermidis (1/5)	neg.	major	Flucloxacillin + Rifampicin 2 weeks then Levofloxacin + Rifampicin 4 weeks	14 months with Staph. hominis
4	fem	30.8	3	4	TKA	Loose-ning after 8 years	2	16	1000	neg.	neg.	2	Staph. epidermidis (2/5)	neg.	major	Flucloxacillin + Rifampicin 2 weeks then Levofloxacin + Rifampicin 4 weeks	no
5	fem.	25.1	3	1	THA	Loose-ning after 10 years	2	< 5	360	neg.	neg.	0	Staph. epidermidis (4/5)	neg.	major	Flucloxacillin + Rifampicin 2 weeks then Levofloxacin + Rifampicin 4 weeks	no
6	fem.	32.9	3	6	THA	Loose-ning after 4 years	1	11	860	Brevi-bacillus brevis (1/5)	neg.	2	Staph. epidermidis (3/5)	positive	major + 5	Vancomycin + Rifampicin 2 weeks then Levofloxacin + Rifampicin 4 weeks	12 months with Staph. epidermi-dis
7	male	26.5	1	0	THA	Loose-ning after 10 years	1	< 5	640	neg.	neg.	0	Staph. caprae (5/5)	neg.	major	Flucloxacillin + Rifampicin 2 weeks then Levofloxacin + Rifampicin 4 weeks	no
8	male	21.9	2	0	THA	Loose-ning after 10 years	2	< 5	1200	neg.	neg.	0	Staph. capitis (5/5) 2 Cutibac-terium agnes (5/5)	positive	major + 3	Vancomycin + Rifampicin 2 weeks then Levofloxacin + Rifampicin 4 weeks	14 months with Staph. capitis
9	male	28.9	2	2	THA	Loose-ning after 9 years	1	< 5	880	neg.	neg.	0	Staph. epidermidis (5/5)	neg.	major	Flucloxacillin + Rifampicin 2 weeks then Levofloxacin + Rifampicin 4 weeks	no
10	male	23.5	2	1	THA	Stem loose-ning after 6 years	2	< 5	1100	neg.	neg.	0	Staph. epidermidis (4/5) Staph. capitits (2/5)	neg.	major	Flucloxacillin + Rifampicin 2 weeks then Levofloxacin + Rifampicin 4 weeks	no
11	fem.	29.6	2	1	THA	Loose-ning after 8 years	5	< 5	480	neg.	neg.	0	Staph. epidermidis (2/5) Cutibac-terium agnes (2/5)	neg.	major	Vancomycin + Rifampicin 2 weeks then Levofloxacin + Rifampicin 4 weeks	4 months with Staph. epidermidis

*Pat.* Patient, *fem.* Female, *BMI* Body mass index, *ASA* Grading of patients for surgical procedures of the American Society of Anesthesiologists [29], *CCI* Charlson Comorbidity Index [30], *WBC* White blood cell count., *y* Years, *neg.* Negative

All expected aseptic hip revisions (118) were performed with cementless hip implants and the expected aseptic knee revisions (214) with cemented revision implants using antibiotic loaded cement with gentamycin and clindamycin (Copal, Heraeus Medical, Hanau, Germany). All of these patients received an intravenous antibiotic prophylaxis of 24 h with a second generation cephalosporin (Cefuroxim). Out of these, all 11 patients with unexpected intraoperative positive PJI (type 1 infection) were given an additional antibiotic therapy of 6 weeks according the susceptibility of the microorganisms immediately after their detection (Table 1).

Of the 508 patients 477 were followed at least 2 years (37.9 ± 16.7 months), (all 178 patients with PJI, including the 11 type 1 infections). According to Masri et al. [31] and Zimmerli et al. [32], a patient could be judged infection-free at follow-up if he or she was free of clinical signs for infection (fever, local pain, redness, warmth, sinus tract infection), and had a CRP level less than 10 mg/L.

### Statistical analysis

Statistical analysis was performed using IBM SPSS Statistics for Windows (version 20.0, IBM Corp., Armonk, NY). Categorial variables were analyzed using a chi-square-test or Fisher’s exact test. All reported *P* values are 2 tailed with an a level < .05 considered significant.

## Results

Of the 11 type 1 infections, 5 (2 TKA, 3 THA) showed reinfection (4, 12, twice 14, and 36 months after revision surgery) with the same microorganism (Table 1). In three cases two bacterial strains were detected (cases 2, 3 and 8 in Table 1), whereby in two cases the second microorganism only grew in extended cultures of one sample and could be interpreted as laboratory contaminants (case 2 and 3 in Table 1). None of the microorganism was multiresistant or a fastidious organism (Table 1). There was no difference in the antibiotic susceptibility of the microorganism between the patients with and without reinfection. The patient with the additional acetabular cup revision did not display any reinfection. This results in a success rate for antibiotic therapy in type 1 infections of at best 54.5% if the patient with the additional cup revision is included. The whole group of 178 patients with PJI had a reinfection rate of 7.3% (13 patients, 5 with type 1 and 8 with type 3 infection (4.8%) (according to Tsukayama et al. [9]) (*p* < 0.001) [22]. The 301 patients with aseptic revision and a minimum follow-up of 2 years had an infection rate of 3.6% (11 patients) (p < 0.001).

## Discussion

By following 11 type 1 infections out of 508 revisions of hip and knee prostheses our hypothesis was tested, that 6 weeks of antibiotic therapy alone do not have a sufficient success-rate in type 1 infection. The reinfection-rate of 45.5% in the current study supports our hypothesis.

The success rate of 54.5% in our study is significantly lower than in all previous, older publications. In the first publication, Tsukayama et al. [9] classified 31 out of 106 (29.2%) PJI cases occurring after total hip endoprostheses as type 1 infections on the basis of a “positive intraoperative culture”. Three patients became reinfected, so that the success rate of antibiotic therapy in these cases was 90%. Segawa et al. [12] unexpectedly found bacteria in 31 of 275 (11%) prosthesis revisions during surgery, in 5 patients in at least two samples (2.1%), but were able to successfully treat 28 (90%), and all 5 of the 5 patients with positive culture in several samples, with antibiotics alone. In Marculesu et al. [11] this type of infection was observed in 3% of cases (16 of 509 prosthesis revisions) and the success rate with antibiotic therapy alone was 93.2% (15 of 16 patients). Barrack et al. [10] found unexpected bacteria in several intraoperative samples from 11 of 692 knee arthroplasty revisions (1.6%). Of these, 2 (18.2%) suffered reinfection. However, the diagnosis of PJI was often not established preoperatively during this time, but was based on intraoperative sampling alone [10–12, 32]; in addition, less than 5 intraoperative tissue samples were obtained in some studies [10]. Moreover, the current criteria for defining a periprosthetic infection were not applied [10–12, 32]. Therefore, we explain the difference between the success rates found in the older studies and our results in this report by the fact that some of the patients in the older studies may have been wrongly classified as PJI because diagnostic procedures were only initiated intraoperatively and the diagnostic methods used were partially inadequate. This resulted in a higher apparent success rate for the therapy of the “type 1 infections” with antibiotic alone.

Our failure rate of 54.5% for the antibiotic therapy of type 1 infections is exactly the same as that seen for a similar situation by Tan et al. [21] and Corró et al. [20] in the two-stage revision of septic prostheses treated with antibiotic alone following a positive bacterial cultures of samples obtained during the second replacement stage.

In our opinion, the low success rate of sole antibiotic therapy is understandable, since up to 14 days can elapse before bacteria can be detected in the cultures of the samples taken intraoperatively [16, 17]. During this time only an antibiotic prophylaxis of 24 h has taken place and the bacteria that remain in situ have already been able to form a new biofilm on the newly implanted prosthesis. The antibiotic therapy that then begins after 14 days would then encounter an already formed biofilm and thus be significantly less effective [16–19]. On the other hand keeping suspected revision cases on antibiotics until cultures are final would lead to a high amount of unnecessary antibiotic treatment. Out of the 332 preoperative diagnostic cases with negative results these 11 cases were 3.3%. This would mean that 96.7% would get unnecessary antibiotic treatment. Therefore exact preoperative diagnostic is essential and in cases of type 1 infection revision surgery may be the treatment of choice with more success.

The study has some limitations. The number of patients in our study is small with 11 patients. However, this small number reflects a good preoperative diagnostic procedure with a low number of false negative results. The preoperative diagnostic procedure for diagnosing PJI in the 508 patients in this study had a sensitivity of 93.8%, a specificity of 97.3%, a positive predictive value of 94.9%, a negative predictive value of 96.7%, and an accuracy of 96.1% [22]. This is in accordance with the results of previous studies using this diagnostic procedure for PJI with accuracies between 93 and 98% [24, 25]. In addition, also in the few previous studies with unexpected intraoperative positive bacterial cultures from several tissue samples, only a small number of patients were assessed (5, 11 and 16 patients, 1.6 to 3% respectively) [10–12]. In our opinion, the significantly lower number of cases with unexpected intraoperative positive bacterial cultures (2.2%) in our study, compared to the study by Tsukayama et al. [9] (29.2%), was due to better diagnostics that involved diagnosis during the preoperative phase, i.e., not only intraoperative diagnostics, as well as our use of modern definitions for PJI. Moreover, our minimum follow-up of 2 years may not be long enough to detect all reinfections in of these 11 patients with type 1 infection. However, if this were the case, this would even result in an even higher reinfection-rate than already determined.

## Conclusions

In summary, antibiotic therapy alone seems to have a significantly lower success rate than previously assumed in the case of unexpected intraoperative bacterial cultures from samples taken during prosthesis revision surgery. This underlines the importance of using preoperative diagnostics that are as accurate as possible in order to exclude a periprosthetic infection prior to a revision operation. It is conceivable that the success rates in type 1 infection can be improved by longer antibiotic treatment, a revision with irrigation, debridement and exchange of mobile components, or even an early one-stage septic exchange of the new implanted prosthesis. However, further studies are required to analyze this.

## Data Availability

We do not wish to share our data, because some of patient’s data regarding individual privacy, and according to the policy of our hospital, the data could not be shared to others without permission.

## Abbreviations

PJI: Periprosthetic joint infection
Pat: Patient
fem: Female
BMI: Body mass index
ASA: Grading of patients for surgical procedures of the American Society of Anesthesiologists
CCI: Charlson Comorbidity Index
WBC: White blood cell count
y: Years
neg: Negative
